# Risk factors and prognostic value of osteoporosis in hospitalized patients with bronchiectasis

**DOI:** 10.1186/s12890-023-02346-2

**Published:** 2023-02-06

**Authors:** Xin Zou, Zhiyi Ma, Xiaohong Liu, Kaijun Zhang, Chenchen Qiu, Rongzhang Liang, Duanli Weng, Lingyan Xie, Xiaoming Cao, Yongquan Wu, Liwen Wen

**Affiliations:** 1 Pulmonary and Critical Care Medicine Department, Longyan First Affiliated Hospital of Fujian Medical University, No. 105 Jiuyibei Road, Longyan, 364000 China; 2 Respiratory Department of Shanghang County Hospital, Longyan, 364000 China; 3 Department of Pharmaceutical Bussiness and Management, Sanming Medical and Polytechnic Vocational College, Sanming, 365000 China

**Keywords:** Osteoporosis, Fragility fracture, Bronchiectasis, Mortality, Comorbidity, Vitamin D, Infections

## Abstract

**Background:**

The risk factors for osteoporosis and its prognostic value in patients with bronchiectasis is not well characterized. We explored the risk factors for osteoporosis and its prognostic impact in hospitalized non-cystic fibrosis bronchiectasis (NCFB) patients in Southeast China.

**Methods:**

This observational cohort study consecutively enrolled 179 hospitalized patients with NCFB bronchiectasis between 2017 and 2021. The risk factors and the impact of osteoporosis on all-cause mortality were assessed.

**Results:**

21.2% (38/179) of hospitalized NCFB patients were diagnosed with osteoporosis. Patients with osteoporosis had more severe symptoms (assessed by chronic airway assessment test, CAT, median 22 vs. 17, *P* = 0.017), poorer quality of life (assessed by St. George Respiratory Questionnaires, SQRC, median 42 vs. 27, *P* = 0.007), more severe disease stage (assessed by bronchiectasis severity index, BSI, median 14 vs. 11, *P* = 0.02), more comorbidities (assessed by Bronchiectasis Aetiology Comorbidity Index, BACI, median 5 vs. 4, *P* = 0.021) than patients without. Age, female sex, anemia, post-infection, and history of regular inhaled corticosteroid treatment were independent risk factors for osteoporosis in those patients. 21 patients (11.7%) died over a median follow-up period of 32 months. The all-cause mortality in NCFB patients with osteoporosis [28.94% (11/38)] was significantly higher than those without osteoporosis [7.09% (10/141)] [hazard ratio (HR) 5.34, 95% confidence interval (CI) 2.26–12.67, *P* < 0.001]. After adjusting for BSI and other confounding factors, osteoporosis was still independently associated with all-cause mortality in hospitalized NCFB patients (HR 4.29, 95% CI 1.75–10.49, *P* < 0.001).

**Conclusions:**

Osteoporosis had an independent effect on all-cause mortality in hospitalized NCFB patients. Management of comorbidities, including bone health, is a critical aspect of treating NCFB patients.

## Background

In the Asian population, non-cystic fibrosis bronchiectasis (NCFB) might be one of the most common but heterogeneous chronic respiratory diseases apart from chronic obstructive pulmonary disease (COPD) and asthma. The prevalence of bronchiectasis in Korea was 9.1% in adults [1]. Although the prevalence of bronchiectasis among the population over 40 years in China was estimated at 1.2% [2], the actual prevalence may be higher because only diagnosed patients were included. Patients with bronchiectasis frequently have other comorbid conditions, which contributes to increased healthcare and socioeconomic burden and worsens the quality of life, exacerbation frequency, lung function, and mortality risk [3]. Similar to asthma and COPD, different comorbidities can significantly impact pulmonary and extrapulmonary manifestations of bronchiectasis, disease severity, prognosis, and therapeutic management. However, despite the high prevalence of comorbid conditions in patients with bronchiectasis, their specific impact on clinical outcomes and the natural history of the disease is not well characterized. Besides, the current clinical guidelines barely include recommendations about managing these conditions due to the paucity of scientific evidence.

Osteoporosis, among the major systemic comorbidities of COPD, is related to systemic inflammatory response, impaired lung function, frequent acute exacerbations, and reduced survival of patients with COPD [4]. Common risk factors for osteoporosis and COPD include age, female sex, early menopause, smoking, alcohol consumption, inadequate vitamin D intake, systemic inflammatory response, low body mass index (BMI), and corticosteroid use [5]. Moreover, as an essential mechanism of osteoporosis, vitamin D deficiency strongly correlates with various health outcomes, including all-cause mortality [6, 7]. The effect of vitamin D on the lungs has a robust scientific rationale; its immunomodulatory, anti-inflammatory, and anti-infective properties have been demonstrated in patients with community-acquired infection, acute respiratory failure, and lung transplantation recipients [8].

Bronchiectasis shares many clinical and pathophysiological characteristics with COPD, including an enhanced local and systemic inflammatory milieu. Besides, recurrent infection leading to permanent bronchi dilation is the primary pathophysiological mechanism of bronchiectasis. In a multicenter cohort study in Europe that evaluated the impact of different comorbidities in bronchiectasis, osteoporosis was the fifth most prevalent comorbidity in NCFB patients [9]. Recent retrospective and cross-sectional studies have also shown a higher prevalence of osteoporosis in bronchiectasis patients compared with the general population [10, 11]. However, the risk factors for osteoporosis and the prognostic value of osteoporosis in bronchiectasis patients are not well investigated.

This study aimed to assess the prognostic impact of osteoporosis in hospitalized NCFB patients and identify the clinical risk factors for osteoporosis in these patients.

## Methods

An observational cohort study of NCFB patients was conducted at the pulmonary and critical care department of the Longyan First Affiliated Hospital of Fujian Medical University in Southeast China from September 2017 to December 2021. Hospitalized patients with a diagnosis of bronchiectasis based on high-resolution computed tomography (HRCT) and respiratory symptoms were enrolled in the study according to the 2012 Expert consensus on diagnosing and treating adult bronchiectasis in China [12]. Exclusion criteria were: (1) cystic fibrosis bronchiectasis; (2) active tuberculosis or nontuberculous mycobacteria infection; (3) malignant tumor; (4) severe immunosuppression (such as solid-organ or bone marrow transplantation, human immunodeficiency virus infection (HIV), or receiving chemotherapy or other immunosuppressive drugs (≥ 20 mg prednisone per day for > 2 weeks); (5) bronchiectasis secondary to interstitial lung disease; and (6) tracheobronchial foreign body. All patients provided written informed consent (Ethical Review of Clinical Research from Longyan First Hospital affiliated with Fujian Medical University).

Clinical data including demographic data, comorbidity (other pulmonary diseases and non-pulmonary diseases), smoking, BMI, clinical symptoms, history of previous hospitalization and exacerbation, possible etiology, radiographic extension (modified Reiff score was calculated as tubular, 1 point; varicose, 2 points; and cystic, 3 points; the total score (six lobes) ranged from 0 to 18 points), FEV1% to predict value (FEV1% pred) and chronic respiratory infection, were collected. The severity of bronchiectasis was assessed using Bronchiectasis Severity Index (BSI) scores [13]. The Chronic airway assessment test (CAT) [14] and St. George's Respiratory Questionnaires were also completed by patients. The Bronchiectasis Aetiology Comorbidity Index (BACI) was calculated according to the derivation of the BACI and point allocation by McDonnell [9]. Patients were followed-up remotely (telephonically or via an electronic form) to record symptoms, death, and treatment details for 3–6 months until June 2022. Macrolides treatment was defined as continuing the medication for at least 3 months.

Osteoporosis was diagnosed based on any of the following criteria: (1) fragile fracture of the hip or vertebra; (2) bone mineral density (BMD) of axial bone or the distal third of the radius measured by Dual-energy X-ray absorption (DEXA) as T < − 2.5; (3) Bone mineral density measurements consistent with low bone mass (− 2.5 < T < − 1.0), with fragile fractures of the proximal humerus, pelvis, or distal forearm [15, 16] Other comorbidities recorded were COPD, asthma, pulmonary artery hypertension (PAH), sinusitis, cardiovascular diseases (CVD), cerebrovascular diseases, peripheral vascular disease (PVD), hypertension (HT), diabetes, hypoxemia, kidney diseases, liver diseases, connective tissue disease (CTD), and gastroesophageal reflux disease (GERD). We recorded COPD as comorbidity similar to other studies [9]; bronchiectasis associated with COPD was defined as the presence of a history of smoking at least 10 pack-years or Biofuel exposure, with airflow obstruction (FEV1/FVC ratio < 0.7) according to the Global Initiative for Chronic Obstructive Lung Disease recommendations.

### Statistical analysis

Statistical analysis was performed using the SAS 9.4 software (SAS Institute, Inc., Cary, NC, USA). Categorical variables were expressed as frequency (%) and compared using the Chi-squared test or Fisher's exact probability method. Continuous variables were expressed by mean ± SD or median (P25, P75). Comparisons among groups were analyzed using the ANOVA or Rank sum tests. Factors with statistically significant differences in the univariate analysis were included in multivariate analysis using Modified Poisson regression analysis to identify risk factors for osteoporosis in hospitalized NCFB patients. Kaplan–Meier curve was constructed to illustrate survival data, and Cox proportional hazard regression model was used for univariate and multivariable mortality analysis to estimate hazard ratios (HRs) and their 95% confidence intervals (CIs). Because the BSI is a comprehensive index including 9 clinical parameters mentioned above (age, BMI, FEV1%pred, history of exacerbation and hospitalization, mMRC score, chronic infection, dilation, imaging) and given the limited sample size of our study, only the BSI score and comorbidities, which were significantly related to survival in univariate analysis, were introduced into the multivariate survival analysis. For all statistical analyses, a *p* value less than 0.05 was considered statistically significant.

## Results

### Basic clinical characteristics

A total of 187 hospitalized patients diagnosed with NCFB who qualified the above criteria were initially enrolled in our study. Four patients were lost to follow-up, and 183 patients completed the follow-up study. Four patients were excluded due to incomplete clinical data. Among the 179 patients who were included in the analysis, 95 were male, and 84 were female. The mean age of the patients was 64.19 ± 14.12 years. The three most common comorbidities were COPD (56/179, 31.28%), hypertension (50/179, 27.93%), PAH (44/179, 24.58%), CVD (41/179, 22.91%), followed by osteoporosis (38/179, 21.23%), anemia (36/179, 20.11%), GERD (33/179, 18.44%), sinusitis (30/179, 16.76%), diabetes ( 24/179, 13.41%), liver disease (22/179, 12.29%), asthma (17/179, 9.50%), connective tissue diseases (15/179, 8.38%), kidney disease (11/179, 6.15%), PVD (9/179, 5.03%), and cerebrovascular disease (7/179, 5.03%). Over a median follow-up of 32 (18, 39) months, 21 patients (11.73%) died (19 died because of respiratory-related disease, and 2 patients died because of non-respiratory-related disease). Twenty-nine patients (16.20%) had chronic infection with pathogenic microorganisms, of which 22 were infected with *Pseudomonas aeruginosa* (PA) (10 were mucoid strains, while 12 were non-mucoid strains), 2 with Escherichia coli, 4 with Aspergillus, and 1 with *Klebsiella pneumoniae*.

### Risk factors for osteoporosis in hospitalized NCFB patients

**Fig. 1:**
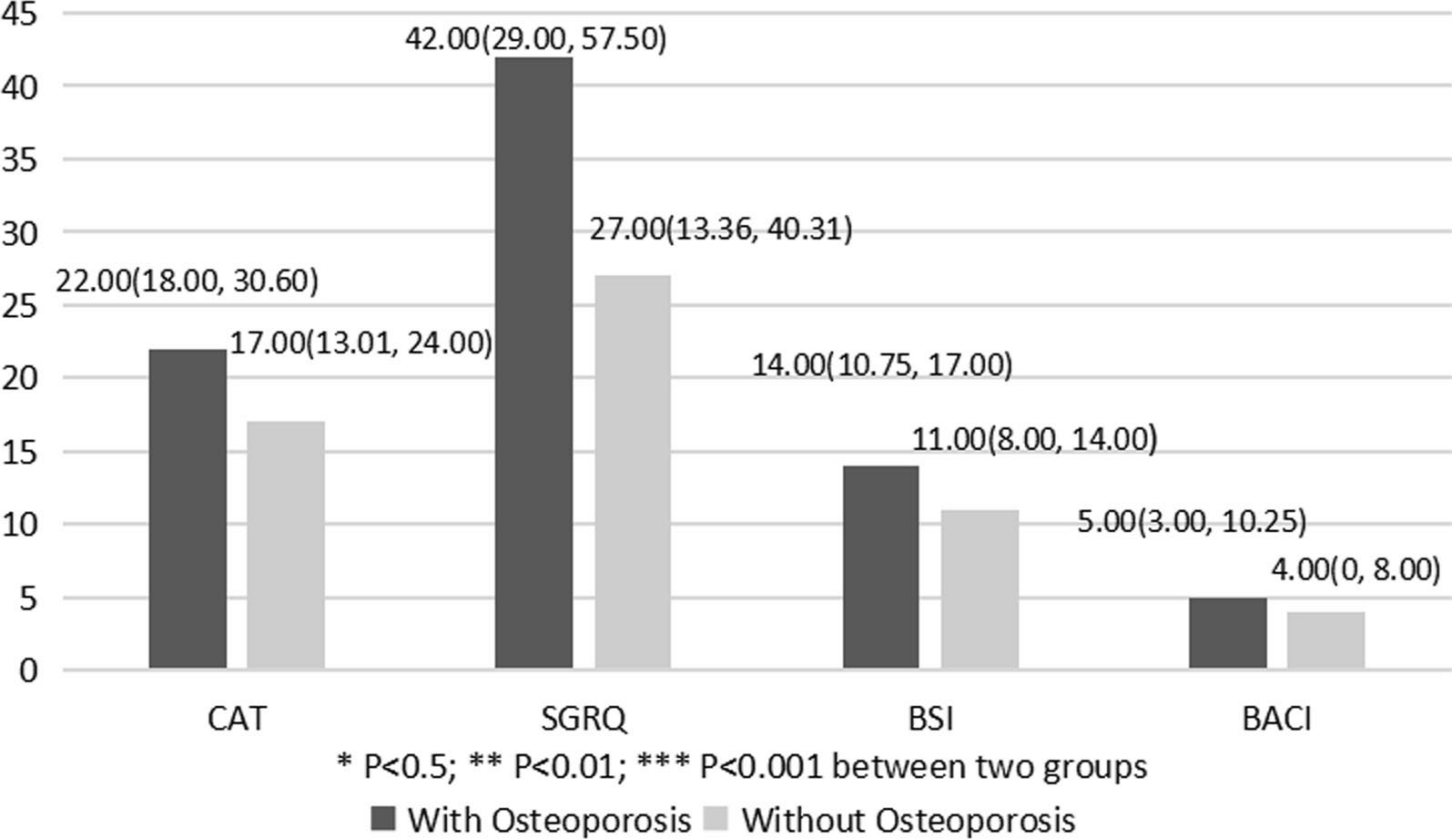
Differences of CAT, SGRQ, BSI, BACI scores between hospitalized NCFB bronchiectasis patients with and without osteoporosis. Data presented as median (interquartile range). Abbreviations: NCFB, non-cystic fibrosis bronchiectasis; CAT, chronic airway assessment test; SGRQ, St. George Respiratory Questionnaires; BSI, Bronchiectasis Severity Index; BACI, Bronchiectasis Aetiology Comorbidity Index

154 patients completed the CAT and SGRQ evaluation. Patients with osteoporosis had significantly higher CAT and SGRQ scores than those without osteoporosis. All the patients completed the BSI and BACI evaluation. The BSI and BACI scores of patients with osteoporosis were also significantly higher than those without osteoporosis (Fig. 1). Patients with osteoporosis were likely to be older, female, suffering from CVD, GERD, and anemia, post-infection, and regular ICS treatment (Table 1). The results of modified Poisson regression analysis showed that age, female sex, anemia, post-infection, and regular ICS treatment were independent risk factors associated with osteoporosis in hospitalized NCFB patients (Table 2).

**Table 1:** Comparison of clinical data in hospitalized NCFB patients with and without osteoporosis

Clinical characteristics	With osteoporosis (n = 38)	Without osteoporosis (n = 141)	*P* value
Age (years)	74.00 (65.00, 78.25)	64.00 (52.50, 73.00)	< 0.001
Sex (male %)	13 (34.21%)	82(58.16%)	0.009
BMI (kg/m^2^)			
< 18.5	14 (36.84%)	41 (29.08%)	0.336
18.5–24	18 (47.37%)	62 (43.97%)	
≥ 24 (%)	6 (23.08%)	38 (26.95%)	
FEV1%pred	48.15 (39.70, 70.00)	60.30 (40.10, 87.45)	0.071
Current or ex-smokers (%)	13 (34.21%)	58 (41.13%)	0.439
Reiff score	8.00 (4.75, 12.00)	8.00 (4.00, 11.00)	0.959
Chronic infection by PA (%)	5 (13.16%)	17 (12.06%)	1.000
Chronic infection by pathogenic microorganisms (%)	7 (18.42%)	22 (15.60%)	0.676
mMRC (> 2 grade) (%)	14 (36.84%)	32 (22.70%)	0.077
Comorbidities			
COPD (%)	15 (39.47%)	41 (29.08%)	0.220
Asthma (%)	5 (13.16%)	12 (8.51%)	0.579
PAH (%)	10. 26 (32%)	34 (24.11%)	0.780
HT (%)	10 (26.32%)	40 (28.37%)	0.802
Diabetes (%)	5 (13.16%)	19 (13.48%)	0.959
Sinusitis (%)	7 (18.42%)	23 (16.31%)	0.757
GERD (%)	13 (34.21%)	20 (14.18%)	0.005
CVD (%)	14 (36.84%)	27 (19.14%)	0.021
Cerebrovascular disease (%)	4 (10.52%)	5 (3.55%)	0.184
PVD (%)	3 (7.89%)	6 (4.26%)	0.622
Kidney disease (%)	2 (5.26%)	9 (6.38%)	1.000
Liver disease (%)	2 (5.26%)	20 (14.18%)	0.227
Anemia (%)	16 (42.11%)	20 (14.18%)	< 0.001
CTD (%)	2 (5.26%)	13 (9.22%)	0.652
Post-infection(%)	14 (36.84%)	30 (21.28%)	0.048
Regular ICS treatment (%)	5 (38.46%)	17 (12.06%)	0.029

Data are presented as frequency (%), mean ± standard deviation, or as median (interquartile range)

*NCFB* non-cystic fibrosis bronchiectasis, *BMI* body mass index, *FEV1%pre* forced expiratory volume in 1 s to predictive value, *PA* Pseudomonas aeruginosa, *mMRC* modified Medical Research Council, *COPD* chronic obstructive pulmonary disease, *PAH* pulmonary artery hypertension, *HT* hypertension, *GERD* gastroesophageal reflux disease, *CVD* cardiovascular diseases, *PVD* Peripheral vascular disease, CTD, connective tissue disease, *ICS* inhaled corticosteroid

**Table 2:** Results of modified Poisson regression analysis showing risk factors associated with osteoporosis among hospitalized NCFB patients

	β-value	SE	Z	RR	95% CI	*P* value
Age	0.03	0.01	2.74	1.03	1.01, 1.06	0.006
Sex (male)	1.02	0.29	3.54	2.77	1.58, 4.87	< 0.001
CVD	0.28	0.30	0.34	1.33	0.74, 2.37	0.339
Post-infection	0.58	0.29	1.99	1.78	1.01, 3.14	0.047
Anemia	0.80	0.26	3.07	2.22	1.33, 3.69	0.002
Regular ICS treatment	1.12	0.26	4.28	3.05	1.83, 5.08	< 0.001
GERD	0.39	0.28	1.39	1.48	0.85, 2.59	0.166
Intercept	− 5.26	0.77	− 6.80	0.01	0.00, − 0.02	< 0.001

Variables with a *P* value < 0.05 in the univariate analysis (as shown in Table 1) were included in the multivariate Modified Poisson regression analysis to determine the risk factors for osteoporosis among hospitalized NCFB patients

*NCFB* non-cystic fibrosis bronchiectasis, *CVD* cardiovascular diseases, *ICS* inhaled corticosteroid, *GERD* gastroesophageal reflux disease, *RR* relative ratio, *95% CI* 95% confidence interval

### Impact of osteoporosis and FF on the mortality of hospitalized NCFB patients

**Fig. 2:**
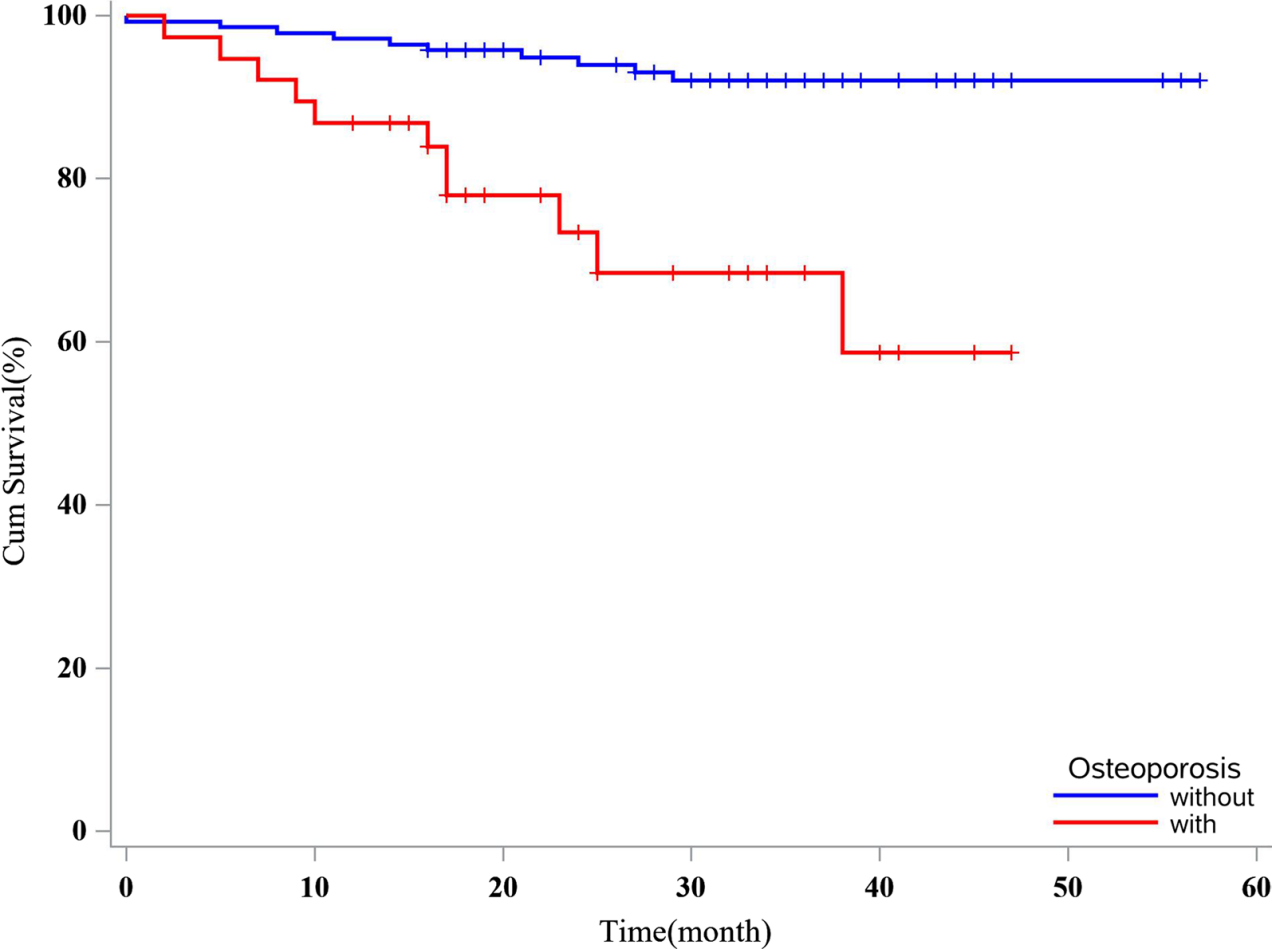
Survival curves of hospitalized NCFB patients with and without osteoporosis after multivariate analysis by Cox proportional hazard model. Abbreviations: NCFB, non-cystic fibrosis bronchiectasis

Over a median follow-up time of 32 (18, 39) months, 21 (11.73%) patients died. The all-cause mortality rate in NCFB patients with osteoporosis was 28.95% (11/38) compared with 7.09% (10/141) in NCFB patients without osteoporosis. Clinical data in our cohort showed that age, BMI, HE in the previous 2 years, AE in the previous 2 years, FEV1%pred, Reiff score, percentage of chronic infection by PA and pathogenic microorganisms, degree of dyspnea (mMRC > 2 grade), and the BSI score were significantly different between survivors and non-survivors (Table 3). Univariate regression analysis showed that the BSI score, comorbidities of PAH, CVD, HT, anemia, cerebrovascular disease, osteoporosis, and macrolides treatment significantly affected the all-cause mortality in our cohort. Multivariate regression showed that the BSI score, comorbid osteoporosis, PAH, CVD, and cerebrovascular disease were independently associated with all-cause mortality in our cohort (Table 4). The all-cause mortality in NCFB patients with osteoporosis [28.94% (11/38)] was significantly higher than those without osteoporosis [7.09% (10/141)] [hazard ratio (HR) 5.34, 95% confidence interval (CI) 2.26–12.67, *P* < 0.001]. After adjusting for BSI and other confounding factors, hospitalized NCFB patients with osteoporosis had an HR of 4.29 (95% CI 1.75–10.49) compared with patients without osteoporosis (survivor curve shown in Fig. 2).

**Table 3:** Comparison of clinical characteristics, BSI, and BACI score of hospitalized NCFB patients between survivors and non-survivors

Clinical characteristics	Survivors (n = 158)	Non-survivors (n = 21)	*P* value
Age (years)	65.00 (55.75, 74.00)	74.00 (58.00, 81.00)	0.033
BMI (kg/m^2^)			
< 18.5 (%)	40 (25.32%)	15 (71.43%)	< 0.001
18.5–24(%)	76 (48.10%)	4 (19.05%)	
≥ 24 (%)	42 (26.58%)	2 (9.52%)	
FEV1%pred	60.65 (40.15, 87.43)	40.00 (30.65, 53.94)	0.001
HE in previous 2 years	1.00 (1.00, 2.00)	2 .00 (1.00,4.50)	0.003
AE in previous 2 years	2.00 (1.00, 3.00)	3.00 (2.00, 8.50)	0.008
Reiff score	7.50 (4.00, 10.00)	12.00 (5.00, 17.00)	0.006
Chronic infection by PA (%)	16 (10.13%)	6 (28.57%)	0.039
Chronic infection by pathogenic microorganisms (%)	19 (12.03%)	10 (47.62%)	< 0.001
mMRC (> 2 grade) (%)	30 (18.99%)	16 (76.19%)	< 0.001
BSI	11.00 (8.00, 14.00)	17.00 (15.00, 19.00)	< 0.001
BACI	4.00 (1.50, 8.00)	8.00 (5.50, 11.00)	< 0.001

Data are presented as frequency (%), mean ± standard deviation, or as median (interquartile range)

*NCFB* non-cystic fibrosis bronchiectasis, *BSI* Bronchiectasis Severity Index, *BACI* Bronchiectasis Aetiology Comorbidity Index, *BMI* body mass index, *FEV1%pre* forced expiratory volume in 1 s to predictive value, *HE* hospitalization with acute exacerbation, *AE* acute exacerbation, *PA* Pseudomonas aeruginosa, *mMRC* modified Medical Research Council

**Table 4:** Univariate regression and multivariate regression for all-cause mortality of hospitalized NCFB patients using Cox proportional hazard model

Basic clinical characteristics	Survivors (n = 158)	Non-survivors (n = 21)	Unadjusted		Adjusted^a^	
			HR (95% CI)	*P* value	HR (95% CI)	*P* value
BSI	11 (8, 14)	17 (15, 19)	1.34 (1.20–1.50)	0.001	1.22 (1.05–1.41)	0.008
Sex (male%) (male-ref)	83 (53.53%)	12 (57.14%)	0.82 (0.34–1.94)	0.647	–	–
Current or ex-smokers (%) (non smokers-ref)	62 (39.24%)	9 (42.86%)	1.19 (0.50–2.82)	0.698	–	–
Comorbidities					–	–
COPD (%)	49 (31.01%)	7 (33.33%)	1.12 (0.45–2.79)	0.801	–	–
Asthma (%)	16 (10.13%)	1 (4.76%)	0.47 (0.06–3.47)	0.802	–	–
PAH (%)	31 (19.62%)	15 (71.42%)	5.13 (2.13–12.39)	< 0.001	3.19 (1.18–8.65)	0.022
HT (%)	39 (24.68%)	11 (52.38%)	3.03 (1.28–7.14)	0.011	–	–
Diabetes (%)	19 (12.02%)	5 (23.81%)	2.24 (0.82–6.11)	0.117	–	–
Sinusitis (%)	29 (18.35%)	1 (4.76%)	0.25 (0.03–1.86)	0.175	–	–
GERD (%)	30 (19.00%)	3 (14.29%)	0.70 (0.21–2.36)	0.560	–	–
CVD (%)	28 (17.72%)	13 (61.90%)	6.29 (2.60–15.19)	< 0.001	2.77 (1.03–7.44)	0.043
Cerebrovascular disease (%)	5 (3.16%)	4 (19.05%)	6.13 (2.05–18.27)	0.001	5.36 (1.62–17.65)	0.006
PVD (%)	9 (5.70%)	0 (0%)	0.05 (0.00–219.42)	0.476	–	–
Kidney disease (%)	9 (5.70%)	2 (9.52%)	1.51 (0.35–6.49)	0.579	–	–
Liver disease (%)	20 (12.66%)	2 (9.52%)	0.79 (0.18–3.39)	0.750	–	–
Anemia (%)	28 (17.72%)	8 (38.10%)	2.71 (1.12–6.54)	0.027	–	–
CTD (%)	13 (8.23%)	2 (9.52%)	1.26 (0.29–5.43)	0.753	–	–
Osteoporosis (%)	27 (17.09%)	11 (52.38%)	5.34 (2.26–12.67)	< 0.001	4.29 (1.75–10.49)	0.001
Post–infection (non post-infection-ref)	36 (22.78%)	8 (38.10%)	2.06 (0.85–4.97)	0.108	–	–
Macrolides treatment (%)	14 (8.86%)	9 (42.86%)	6.08 (2.56–14.45)	< 0.001	–	–

Data are presented as frequency (%), mean ± standard deviation, or as median (interquartile range), unless otherwise stated. a: BSI, PAH, HT, CVD, cerebrovascular disease, anemia, osteoporosis, and macrolides treatment showed a significant association with survival (P < 0.05) in the univariate survival analysis and were entered in the multivariate analysis model. Cox proportional hazard regression (method: Forward LR) showed that BSI, PAH, CVD, cerebrovascular disease, and osteoporosis were independently associated with the all-cause mortality of hospitalized NCFB patients

*NCFB* non-cystic fibrosis bronchiectasis, *BSI* Bronchiectasis Severity Index, *COPD* chronic obstructive pulmonary disease, *PAH*, pulmonary artery hypertension, *HT*, hypertension, *GERD*, gastroesophageal reflux disease, *CVD*, cardiovascular diseases, *PVD*, peripheral vascular disease, *CTD*, connective tissue disease, *ICS*, inhaled corticosteroid, *HR*, hazard ratio, *95% CI* 95% confidence interval

## Discussion

In our study, NCFB patients with osteoporosis were found to have more severe symptoms (CAT score), worse quality of life (SGRQ score), greater disease severity (BSI score), and more comorbidities (assessed by BACI score) than patients without osteoporosis. Age, female sex, anemia, post-infection, and regular ICS treatment were risk factors for osteoporosis in hospitalized NCFB patients. Osteoporosis was also found to be an independent risk factor for all-cause mortality after adjusting for BSI and other confounding factors, which suggests that clinicians should pay attention to the bone health of NCFB patients. To the best of our knowledge, few studies have investigated the effect of osteoporosis on the prognosis of patients with NCFB, especially in the Asian population.

Osteoporosis is a common finding in NCFB patients worldwide. According to a European study (2015), osteoporosis ranks amongst the top five comorbidities associated with NCFB; in this multicenter European NCFB cohort (986 patients), 15.9% of patients had osteoporosis [9]. In a retrospective study based on electronic medical records in Mayo Clinic, Florida, the prevalence of osteoporosis in NCFB patients reached 30% [15]. In another cross-sectional study conducted in Malaga, Spain, 7% of men and 15% of women with NCFB had osteoporosis [11]. A recent study in China showed a much higher prevalence of osteoporosis in Asian patients with NCFB (70%) [17]. The considerable differences in the reported incidence of osteoporosis in NCFB patients may be attributable to differences in the study population, methods, and data collection methods. Our study found a moderate probability of osteoporosis in hospitalized NCFB patients (21.23%) compared with previous studies; this may be because of a higher proportion of males in our cohort and the fact that not all NCFB patients in our study underwent bone mineral density (BMD) test, which may have underestimated the prevalence in some asymptomatic patients.

The harmful impact of osteoporosis in patients with chronic pulmonary diseases has been widely investigated, especially in COPD and asthma. Vertebral fractures (VF) and bone loss in elderly postmenopausal women were closely related to restrictive lung dysfunction [18]. The decrease in bone density in COPD patients was closely related to the degree of emphysema [19] and the severity of airflow limitation [20]. A meta-analysis showed that osteoporosis and VF significantly increase the mortality risk and decrease pulmonary function among patients with COPD [4]. Among asthmatic patients, osteopenia, osteoporosis, and pathologic fractures were identified as risk factors for emergency department visits [21]. In cystic fibrosis (CF) patients with end-stage lung disease, CF-related bone diseases such as decreased bone mineral density were also commonly detected [22]. There has even been a dicey combination of malignancy (lung cancer) and osteoporosis [23].

The underlying mechanisms of the association between bronchiectasis and osteoporosis are not well characterized. Corticosteroid therapy for comorbidities of COPD or asthma may play a significant role in this respect. In addition, they might share common risk factors, including old age, lower BMI, sex, systematic inflammation, and Vitamin D deficiency. Beyond the classical risk factors mentioned above, especially in tuberculosis-endemic Asian regions, tuberculosis significantly increases the incidence of osteoporosis and osteoporotic fracture in NCFB patients [24]. In our study, too, post-infection (including tuberculosis and other pathogens) were independent risk factors for osteoporosis in hospitalized NCFB patients after adjusting for age, sex, and regular ICS treatment. Systematic inflammation, vitamin D deficiency, and the non-calcemic effect of vitamin D and 1,25(OH)_2_ D on the organs may mediate the association between osteoporosis and pulmonary diseases [25–28]. Besides, genetic polymorphisms in vitamin D receptor (*VDR*) and vitamin D-binding protein (VDBP) may also play a role in the pathogenesis of lung diseases [26, 29].

In our study, anemia was found to be another independent risk factor for osteoporosis in patients with bronchiectasis, similar to several previous studies that described a strong association between bone health and anemia [11, 30]. There are several mechanisms by which clinically detectable anemia could signal bone fragility. The first potential mechanism is impairment of bone cells in providing a supportive microenvironment required to maintain erythropoiesis. Moreover, other scholars have detected that anemia or iron deficiency can increase hematopoietic lineage cells (including osteoclasts) and marrow expansion by decreasing bone volume. Moreover, anemia can affect bone strength by altering the mechanical and loading properties of bone marrow. Another potential mechanism is increased erythropoietin levels, which stimulates bone marrow erythropoiesis and induces bone loss [30]. Although several studies have shown a strong relationship between bone health and anemia in individuals, more work is required to elucidate the relationship between hemoglobin (HGB) levels and bone density, and to evaluate the utility of HGB measurement as a signal for osteoporosis screening.

The causal relationship between osteoporosis and pulmonary diseases and the underlying molecular mechanisms are also not yet fully elucidated. In addition to the common risk factors such as old age, smoking, low BMI, and reduced physical activity, systemic or long-term inhaled glucocorticoid therapy is an established reason for the greater susceptibility of COPD and asthma to develop osteoporosis or fragility fracture (FF) [21]. An increasing number of studies have demonstrated that systemic inflammation is a potential cause of pulmonary disease-associated osteoporosis [19, 31, 32]. Moreover, studies have shown a vital role of vitamin D in mediating the link between osteoporosis and pulmonary diseases [33]; the potential non-calcemic effects of vitamin D have evoked the interest of many researchers. Studies have demonstrated the potential link between vitamin D insufficiency or deficiency and COPD pathogenesis, progression, exacerbations, and comorbidities [25]. In our study, osteoporosis was an independent risk factor for mortality in hospitalized NCFB patients. To the best of our knowledge, this is the first study to directly demonstrate the adverse impact of osteoporosis in NCFB patients of Asian ethnicity. Recently, several studies have investigated the effect of osteoporosis in patients with bronchiectasis. Osteoporosis was the fifth most prevalent comorbidity in the BACI derivation cohort, but it was not associated with a significant risk of 5-year mortality [9]. In a cross-sectional study of 123 patients with bronchiectasis, FEV1, body composition, muscle strength, and bone remodeling markers were associated with a lower BMD [11]. In another retrospective cross-sectional study of NCFB patients, BMD showed a significant negative correlation with the lowest SPO_2_, the change of SPO2%, and distance-saturation product during the 6-min walk test; the BMD also showed a decrease with the increase of severe exacerbations per year [17].

## Limitations

Some limitations of our study should be considered while interpreting the results. First, this was a single-center study and the study population comprised of hospitalized patients, indicating relatively severe disease. Although studies conducted in Europe did not find a significant contribution of osteoporosis to the mortality of NFCB patients [9], different ethnic groups and disease spectrum in different regions may lead to different risk factors. Second, this was an observational study, and the relevant comorbidities were captured from the medical history or examination findings in the electronic medical records. Thus, the possibility of reporting bias cannot be ruled out and some comorbidities may have been missed if patients had no symptoms. Moreover, although we had excluded patients who had malignant tumor or severe immunosuppression (such as oral glucocorticoids treatment), concurrent medication (such as proton pump inhibitors or insulin sensitizing agents) or follow-up intervention may have influenced bone mineral density. Further interventional studies are required to validate the cause-effect relationship between the two diseases. Third, we did not test the level of 1,25(OH)_2_ D or any other factor that reflects the association between osteoporosis and NCFB. More extensive studies are required to determine the exact impact of osteoporosis in Asian NCFB patients and unravel the associated mechanisms.

## Conclusions

Age, female sex, history of infection, anemia, and regular ICS treatment were risk factors associated with osteoporosis in hospitalized NCFB patients from Asia. Our study indicates that comorbid osteoporosis in hospitalized NCFB patients is an independent risk factor for all-cause mortality. Clinicians should pay attention to bone health in patients with chronic pulmonary disease, including bronchiectasis. Further studies are required to investigate the potential mechanism between those two diseases.

## Data Availability

The data used to support the findings of this study are available from the corresponding author upon request.

## Abbreviations

NCFB: Non-cystic fibrosis bronchiectasis
FF: Fragility fracture
BMI: Body mass index
PA: Pseudomonas aeruginosa
mMRC: Modified Medical Research Council
FEV1%pre: Forced expiratory volume in 1 s to predictive value
BSI: Bronchiectasis Severity Index
BACI: Bronchiectasis Aetiology Comorbidity Index
COPD: Chronic obstructive pulmonary disease
PAH: Pulmonary artery hypertension
HT: Hypertension
GERD: Gastroesophageal reflux disease
CVD: Cardiovascular diseases
PVD: Peripheral vascular disease
CTD: Connective tissue disease
ICS: Inhaled corticosteroid
CAT: Chronic airway assessment test
SGRQ: St. George Respiratory Questionnaires
RR: Relative ratio
HR: Hazard ratio
95% CI: 95% Confidence interval
BMD: Bone mineral density
DEXA: Dual-energy X-ray absorptiometry
VF: Vertebral fractures
VDR: Vitamin D receptor
VDBP: Vitamin D-binding protein
HGB: Hemoglobin
FF: Fragility fracture
